# ADAR1 Alleviates Inflammation in a Murine Sepsis Model via the ADAR1-miR-30a-SOCS3 Axis

**DOI:** 10.1155/2020/9607535

**Published:** 2020-03-24

**Authors:** Zhou Shangxun, Li Junjie, Zhao Wei, Wang Yutong, Jia Wenyuan, Liu Shanshou, Wang Yanjun, Wang Qianmei, Feng Zhusheng, Yu Chaoping, Zhuang Ran, Yin Wen, Huang Yang

**Affiliations:** ^1^Department of Emergency, Xijing Hospital, Fourth Military Medical University, #169 Changle Xi Road, Xi'an, Shaanxi Province, China 710032; ^2^Department of Immunology, Fourth Military Medical University, #169 Changle Xi Road, Xi'an, Shaanxi Province, China 710032

## Abstract

Adenosine deaminase acting on double-stranded RNA 1 (ADAR1) mediates adenosine-to-inosine (A-to-I) RNA editing events. ADAR1 is highly expressed in “septic” macrophages and in small intestinal tissues of mice with sepsis. Overexpression of ADAR1 suppresses inflammation and intestinal damage. However, the specific underlying mechanism is unclear. This study was conducted to explore how microRNA (miRNA) regulates the anti-inflammatory mechanism of macrophages following ADAR1 upregulation. A murine sepsis model was established by cecal ligation and puncture (CLP). Mice were randomly assigned to sham, CLP, and CLP+ADAR1 groups. Hematoxylin and eosin (HE) staining and fluorescence isothiocyanate-dextran were used to evaluate intestinal injury and permeability. Quantitative reverse transcription-polymerase chain reaction (qRT-PCR), western blotting, and Luminex assays were performed to detect changes in the expression of inflammatory cytokines. Adenoviruses were used to express ADAR1 in RAW 264.7 cells. Ribonucleoprotein immunoprecipitation analysis was conducted to detect the binding of ADAR1 and miRNAs. A dual-luciferase reporter assay was used to detect the binding of miRNAs and regulatory factors. We observed that ADAR1 significantly increased the expression of suppressor of cytokine signaling 3 (SOCS3) in macrophages and reduced the expression of interleukin-6 in macrophages and the serum, thereby reducing intestinal permeability and mucosal injury in mice with sepsis. The RNA-ribonucleoprotein immunoprecipitation binding assay and qRT-PCR demonstrated a direct interaction between ADAR1 and pri-miR-30a. The luciferase assay demonstrated that SOCS3 was significantly inhibited by miR-30a-5p, the mature product of miR-30a. Thus, ADAR1 exerts a protective effect against sepsis by reducing inflammation and organ damage via the ADAR1-miR-30a-SOCS3 axis.

## 1. Introduction

Sepsis is a global healthcare problem caused by the presence of infectious factors. It is a common complication in severe trauma, shock, infection, and other critical illnesses and affects 30 million people annually [1]. Even survivors risk lifelong complications; approximately half of sepsis survivors experience long-term psychological and physiological effects [2]. Sepsis is associated with infectious agents, hypersensitive cascades of severe tissue damage, and excessive release of inflammatory mediators. Release of the proinflammatory cytokines interleukin- (IL-) 1*β* and IL-6 and immunomodulatory cytokine IL-10 by innate immune cells including macrophages, granulocytes, and natural killer (NK) cells during sepsis is well documented [3]. The responses of macrophages to cytokines and chemokines eventually intensify the inflammatory response, thereby leading to septic shock and even death [1].

Adenosine deaminase acting on double-stranded RNA 1 (ADAR1) mediates adenosine-to-inosine (A-to-I) RNA editing events. This RNA editing function is an epigenetic event resulting in posttranscriptional modifications of pre-mRNA [4]. It is ubiquitous in mammals, with millions of A-to-I editing events identified in the human transcriptome to date. However, in mammals, most A-to-I edits occur in noncoding regions, particularly within the SINE family of retrotransposons that form long double-stranded RNAs; ADAR1 is the main enzyme responsible for editing these duplicates [5]. In tumor therapy, loss of ADAR1 function in tumor cells removes a checkpoint that normally restrains sensing of interferon-inducible double-stranded RNA, leading to enhanced tumor inflammation and heightened interferon sensitivity [6]. ADAR1 plays a critical role in regulating innate immune activation by suppressing endogenous RNA sensing because ADAR1 deficiency causes sterile inflammation and tissue injury [7]. Nakahama et al. reported that inhibition of MDA5 activation by ADAR1 is essential for thymocyte maturation and for preventing colitis [8, 9]. However, the biological significance of these changes is not completely understood. Our previous studies demonstrated that ADAR1 suppresses miR-21 maturation, upregulates the expression of forkhead box protein O1 (Foxo1), promotes interleukin- (IL-) 10 expression, modulates macrophage M2 polarization, and alleviates allogeneic graft rejection [10]. In addition, overexpression of ADAR1 can reduce the mortality of mice with infection, and suppression of ADAR1 in mice with sepsis significantly enhanced inflammation and intestinal damage, whereas overexpression of ADAR1 significantly reduced damage and inflammation, thus improving the survival of mice with sepsis [11].

Suppressor of cytokine signaling 3 (SOCS3) is a feedback inhibitor of the Janus kinase (JAK)/signal sensor and transcriptional activation 3 (STAT3) signaling pathway, which mediates the signal transduction of many cytokines and has been implicated in multiple infectious and inflammatory diseases [12]. Studies have shown that SOCS3 is regulated by miR-30a and miR-30a-5p [13–15].

Here, we observed that ADAR1 regulates SOCS3 expression by controlling miR-30a maturation, thereby modulating the inflammatory response in the acute stage of sepsis and ameliorating its symptoms.

## 2. Materials and Methods

### 2.1. Murine Sepsis Model

Male, 6~8-week-old C57BL/6 mice (20-25 g) were obtained from the animal center of the Fourth Military Medical University (Xi'an, China) and were housed under standard laboratory conditions. Mice were randomly divided into sham (*n* = 3), cecal ligation and puncture (CLP; *n* = 3), and CLP+ADAR1 (*n* = 3) groups. Sepsis was induced via CLP as described previously [16]. The hair on the abdomen was clipped, and a small midline abdominal incision (1 cm) was generated. Two punctures were made through the cecum with a 21-gauge needle and feces were extruded from the resulting orifices. The abdominal incision was closed in two layers. Sham mice were treated identically, except that the cecum was neither ligated nor punctured. All mice were administered 1 mL normal saline subcutaneously after surgery to compensate for fluid loss. CLP mice were treated with the negative control adenovirus or ADAR1 overexpression adenovirus (GenePharma, Shanghai, China). In total, 1 × 10^8^ plaque formation units of adenoviruses in 200 *μ*L phosphate-buffered saline were administered via the epicanthic vein after CLP. Serum inflammatory cytokine levels were measured by enzyme-linked immunosorbent assay after 24 h. To assess the extent of intestinal injury, fluorescein isothiocyanate- (FITC-) dextran gavage was performed on mice at 24 h after CLP, and the small intestine samples were collected for histological analysis. All experiments in this study were described in a protocol (No. 20190305) approved by the Welfare and Ethics Committee, Laboratory Animal Center, Fourth Military Medical University.

### 2.2. Detection of Intestinal Permeability Using FITC-Dextran

After establishing sepsis, gavage with 4000 Da FITC-dextran (600 mg/kg body weight, 80 mg/mL in phosphate-buffered saline, Sigma-Aldrich, St. Louis, MO, USA) was performed, and blood sample was collected after 1 h. The plasma FITC-dextran concentration was determined using a fluorescence spectrophotometer at an emission wavelength of 520 nm [17–19]. FITC-dextran infiltration in the tissue was further observed by fluorescence microscopy (EVOS FL, Life Technologies, Carlsbad, CA, USA).

### 2.3. Histology

Intestinal tissues were embedded in paraffin, sectioned to 5 *μ*m thickness, and stained with hematoxylin and eosin (HE). Sections were examined and scored by pathologists blinded to each other's observations. Pathological damage to the intestinal tissues in each group was analyzed semiquantitatively: 0 indicated normal mucosal villi; 1 indicated the subepithelial space above the villi; 2 indicated moderate elevation of the superior cortex of the lamina propria; 3 indicated large epithelial uplift on both sides of the villi (a few tips may fall off); 4 indicated villus shedding, lamina propria exposure, and telangiectasia; and 5 indicated lamina propria calving, hemorrhage, and ulcer [20–22].

### 2.4. Quantitative Reverse Transcription-Polymerase Chain Reaction (qRT-PCR)

Total RNA was extracted from RAW 264.7 cells using the TRIzol reagent according to the manufacturer's protocol (Invitrogen, Carlsbad, CA, USA). First-strand cDNA was synthesized in a volume of 20 *μ*L using 1 *μ*g total RNA and PrimeScript® RT reagent (Takara, Shiga, Japan). RNA was quantified at 260 nm using a spectrophotometer, and only samples with an OD 260/280 ratio between 1.8 and 2.0 were used. CD226 and *β*-actin mRNA expressions were analyzed by RT-PCR with a SYBR Green kit (Takara) to simultaneously assay multiple genes. The primer pairs were designed by Tsingke Biological Technology (Beijing, China) and are shown in Table 1.

### 2.5. Luminex Assay and Magnetic Bead Kits

After successfully establishing the sepsis model, mice were anesthetized using isoflurane gas. A 1 mL syringe was held in the right hand with the bevel tip facing upwards, and the heart was pierced from the strongest beating point at the junction of the xiphoid and the left costal arch at an angle of approximately 15-30 degrees relative to the abdomen. It was inserted approximately 0.5 cm-1 cm until blood was observed flowing into the syringe. The syringe was continued to be held in that position, and the plunger was withdrawn at a uniform speed to collect as much blood as possible. Approximately 1 mL of blood was drawn from each mouse. The collected blood was transferred to a clean EP tube, kept at 4°C for 24 h, and centrifuged at 3,000 rpm for 10 min to obtain the serum. Approximately 200 *μ*L of serum was obtained from each mouse. The levels of 10 cytokines in the serum samples were estimated using a multiplex magnetic bead-based Luminex assay (R&D Systems, Minneapolis, MN, USA). The beads were added to 96-well plates and washed with a Tecan magnetic plate washer. Fifty microliters of the prepared standard, sample, and blank were added to the corresponding wells, sealed with film, and placed on a magnetic plate shaker at 850 rpm for 2 h in the dark at room temperature (15–25°C). The sample was discarded, and the plate was subjected to three consecutive washes using an automated washer. Fifty microliters of diluted biotin cocktail (Invitrogen) was added per well, sealed, and placed on a flat-panel shaker. The plate was oscillated at 850 rpm for 1 h in the dark at room temperature. Next, the biotin antibody cocktail was added and washed three times with the automated washer, followed by an addition of 50 *μ*L diluted streptavidin-PE (Thermo Fisher Scientific, Waltham, MA, USA) to each well, and the plate was sealed with a film and oscillated on a flat shaker at 850 rpm for 30 min in the dark at room temperature. The samples were then washed three times using the automated washer. One hundred microliters of wash buffer was added per well, and the plate was sealed with a film and placed on the flat shaker oscillating at 850 rpm for 2 min in the dark at room temperature. The plates were read using Luminex 200 instrument.

### 2.6. Cell Culture and Treatment

The RAW 264.7 macrophage cell line was purchased from the cell culture center of the Chinese Academy of Medical Sciences (Beijing, China). The cells were cultured in Roswell Park Memorial Institute 1640 medium (Gibco BRL, Grand Island, NY, USA) supplemented with 10% fetal bovine serum (Gibco BRL) at 37°C in a humidified atmosphere of 5% CO_2_. The adherent cells were passaged every 2–3 days. Macrophages were activated using lipopolysaccharide (LPS) to establish an *in vitro* “sepsis” model [23]. Briefly, 1 × 10^6^ macrophages were inoculated into a 60 mm culture dish (Corning, Inc., Corning, NY, USA) and treated with 200 ng/mL LPS (Sigma-Aldrich). Western blotting was performed on cells lysed with radioimmunoprecipitation assay buffer supplemented with complete protease inhibitor cocktail (Roche, Basel, Switzerland). qRT-PCR was performed on RNA isolated from the cells using TRIzol reagent (Invitrogen).

### 2.7. Western Blotting

RAW 264.7 cells were grown to 80-90% confluence in 60 mm dishes. Total cellular proteins were extracted in radioimmunoprecipitation assay buffer containing 1% phenylmethylsulphonyl fluoride and 1% protease inhibitor. Protein concentrations were quantified using a Bicinchoninic Acid Protein Assay Kit (Pierce, Rockford, IL, USA). Samples were subjected to sodium dodecyl sulfate-polyacrylamide gel electrophoresis, and the proteins were transferred onto a polyvinylidene fluoride membrane for analysis. The blots were blocked with 5% skim milk in Tris-buffered saline-Tween 20 for 1 h at room temperature and then incubated with mouse anti-ADAR1 or rabbit anti-SOCS3 monoclonal primary antibody overnight at 4°C. After washing, a 1 : 2000 dilution of horseradish peroxidase-labeled secondary antibody (Pierce) was applied and incubated for 1 h at room temperature. The blots were visualized using enhanced chemiluminescence-Plus reagent (GE Healthcare, Little Chalfont, UK). An anti-*β*-actin antibody was used to confirm equal protein loading.

### 2.8. Cell Transfection

The adenoviruses constructed for overexpressing *ADAR1* (Ad-O/E-ADAR1), short hairpin RNA mediating *ADAR1* knockdown (Ad-shADAR1), and negative control (Ad-NC) were designed and packaged by GenePharma. RAW 264.7 cells were seeded into six-well plates. At 70-80% confluence, the cells were transiently transfected with miR-30a-5p mimic, miR-30a-5p inhibitor, or negative control (NC) siRNAs (Tsingke Biological Technology) using Lipofectamine 3000 transfection reagent (Invitrogen) according to the manufacturer's instructions. Gene silencing was observed by qRT-PCR at 48 h posttransfection.

### 2.9. RNP Immunoprecipitation

Vienna-RNA Web Services is an open-source collaborative secondary RNA structure analysis software available from the Theoretical Biochemistry Group (http://rna.tbi.univie.ac.at/). To assess the association of endogenous ADAR1 with endogenous pri-miR-30a RNA, we performed immunoprecipitation of RNP complexes as described previously [24]. Twenty million RAW 264.7 mouse macrophage cells were collected and lysed. The lysates were subjected to immunoprecipitation for 4 h at room temperature in the presence of 30 mg anti-ADAR1 or anti-mouse IgG. Coimmunoprecipitated RNA was reverse transcribed, followed by qRT-PCR analysis to measure the abundance of pri-miR-30a and U6 RNAs.

### 2.10. Dual-Luciferase Reporter Assay

Wild-type (WT) mRNA-SOCS3 (SOCS3 WT: 5′-3′:TGTTTTGAATAATGTTTACAATTTGCCTCAATCACT), which can bind to miR-30a-5p, was amplified by PCR and inserted into the pmirGLO dual-luciferase miRNA target expression vector (GenePharma). The mutant-type (MUT) mRNA-SOCS3 (SOCS3 MUT: 5′-3′:TGTTTTGAATAAACAAATGTATTTGCCTCAATCACT) was inserted into the reporter vector and transfected as the NC. These vectors were cotransfected with miR-30a-5p mimic or mimic-NC into cells using Lipofectamine 3000 reagent. At 24 h posttransfection, the dual-luciferase reporter assay system (Promega, Madison, WI, USA) was used to determine luciferase activity.

### 2.11. Statistical Analyses

Data were analyzed using the GraphPad Prism software (v.6.0; GraphPad, Inc., La Jolla, CA, USA). Student's *t*-test and one-way analysis of variance were used. A *p* value < 0.05 was considered statistically significant.

## 3. Results

### 3.1. Overexpression of ADAR1 Alleviates Sepsis-Associated Intestinal Damage and Systemic Inflammatory Response

**Figure 1:**
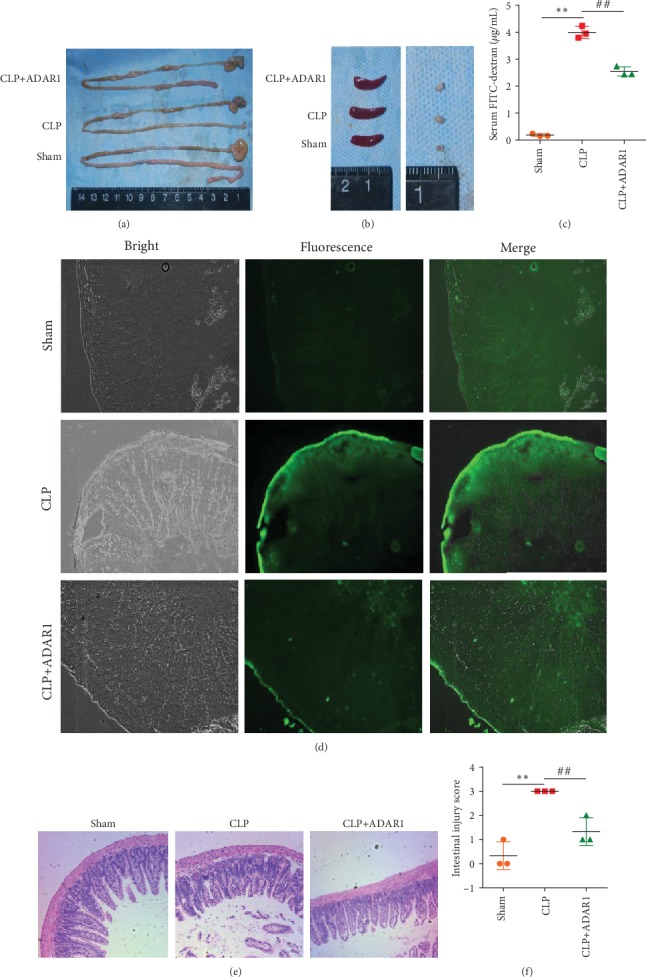
Ad-O/E-ADAR1 ameliorates sepsis in a mouse model. (a, b) The small intestine, spleen, and lymph nodes were dissected to observe morphologic changes in the gross specimens. (c, d) Intestinal leakage assayed by FITC-dextran gavage (100x) and measurements of FITC-dextran concentrations in the blood. (e, f) Representative images of villar injury in the small intestine and scored by HE staining (100x). CLP vs. sham: ^∗^*p* < 0.05, ^∗∗^*p* < 0.01; CLP+ADAR1 vs. CLP: ^#^*p* < 0.05, ^##^*p* < 0.01.

Macroscopic observation showed that the intestinal tissues of mice in the sham-operated group had good elasticity and no pneumatosis or bleeding. The small intestines of the animals from the CLP and CLP+ADAR1 groups showed only slight hyperemia (Figure 1(a)). The spleens and lymph nodes of the CLP group were larger than those of the sham group, whereas those of the CLP+ADAR1 group were smaller (Figure 1(b)). FITC-dextran cannot penetrate the gastrointestinal barrier under physiological conditions; it can be used to evaluate injury to the small intestine, because the resulting inflammation leads to increased mucosal permeability. Mice with sepsis were inoculated with FITC-dextran and dissected after 1 h. As shown in Figures 1(c) and 1(d), in the CLP group, fluorescence was observed in all layers of the small intestine, with the highest intensity in the basement membrane. In the CLP+ADAR1 group, the intensities were significantly lower than those in the CLP group (Figure 1(d)). The serum FITC-dextran level in the CLP+ADAR1 group was significantly lower than that in the CLP group (*p* < 0.001). Thus, ADAR1 exerted a protective effect on the intestinal mucosal barrier, which assisted in maintaining intestinal permeability and function.

Light microscopic observation showed that the intestinal tissues of the sham control group were clear and normal, with an intact and continuous epithelium and intact but irregular villar continuity. Further, the mucosa, submucosa, and lamina propria exhibited no congestion, edema, or rupture. In the CLP group, extensive villar shedding was observed, with the lamina propria and submucosa showing mild edema. The villi of the CLP+ADAR1 group showed good continuity and regular arrangement without obvious shedding, hyperemia, or edema (Figure 1(e)). The intestinal injury scores of the CLP+ADAR1 group were significantly lower than those of the CLP group (Figure 1(f), *p* = 0.008).

### 3.2. Effect of ADAR1 on Systemic Inflammation in Septic Mice

**Figure 2:**
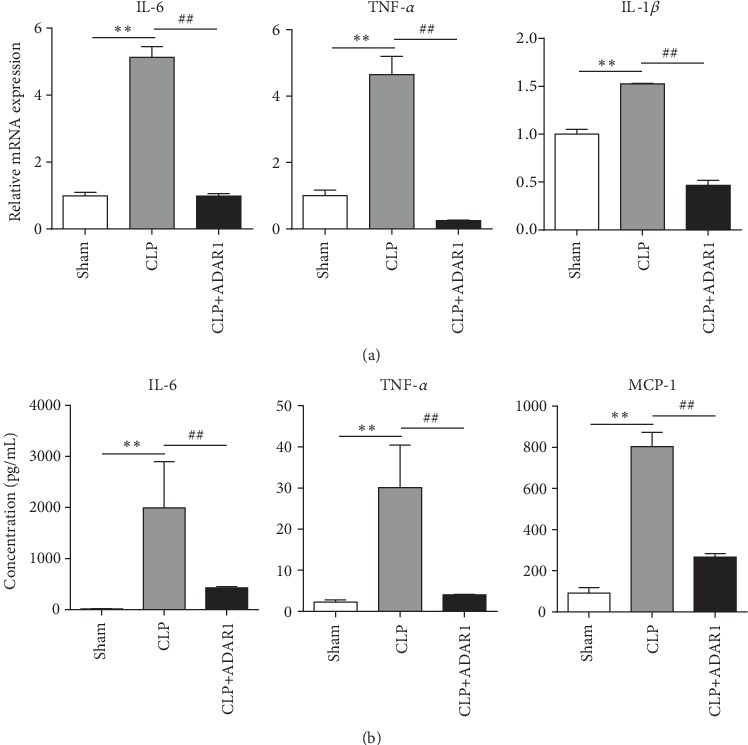
Overexpression of ADAR1 reduces systemic inflammation in mice with sepsis. (a) Expression of IL-6, TNF-*α*, IL-1*β*, and IL-4. (b) Luminex detection of inflammatory factors IL-6, TNF-*α*, MCP-1, and IL-4 in mouse serum. CLP vs. sham: ^∗^*p* < 0.05, ^∗∗^*p* < 0.01; CLP+ADAR1 vs. CLP: ^#^*p* < 0.05, ^##^*p* < 0.01.

The expression levels of IL-6, tumor necrosis factor- (TNF-) *α* and IL-1*β* in the spleens of the CLP group were significantly elevated, whereas the levels of these proteins were remarkably decreased following ADAR1 overexpression (Figure 2(a)). We further detected the circulating levels of relevant inflammatory factors to determine the anti-inflammatory effects of ADAR1. Serum concentrations of IL-6, TNF-*α*, and monocyte chemoattractant protein- (MCP-) 1 in the CLP group were significantly elevated, but this effect was eliminated following ADAR1 overexpression (Figure 2(b)). These data suggest that ADAR1 significantly inhibited activation of the systematic inflammatory response in the acute stage of sepsis; its effects on IL-6 and TNF-*α* were the most dramatic. Furthermore, MCP-1 in the serum was notably decreased in the CLP+ADAR1 group than in the CLP group, suggesting that ADAR1 can ameliorate inflammation via monocyte/macrophage-mediated signaling pathways.

### 3.3. ADAR1 Regulates Expression of IL-6 in Macrophages via SOCS3

**Figure 3:**
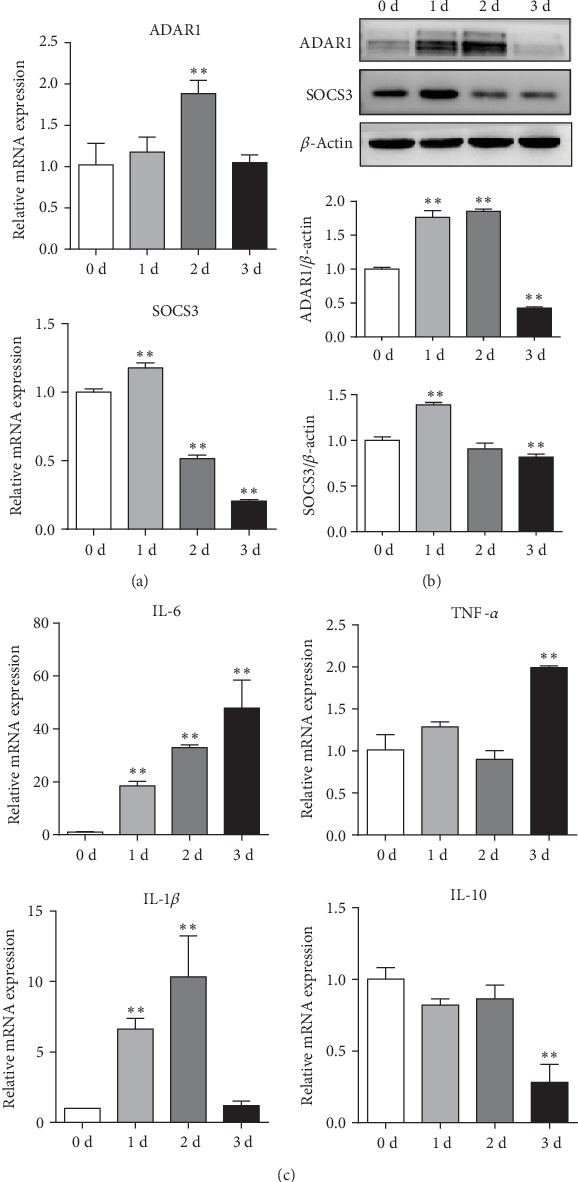
ADAR1 regulates the expression of IL-6 via SOCS3 in RAW 264.7 cells. (a) ADAR1 and SOCS3 mRNA and (b) protein expressions in RAW 264.7 cells after stimulation with LPS. (c) Expression of mRNA for inflammatory factors IL-6, TNF-*α*, IL-1*β*, and IL-10. 1, 2, and 3 days vs. 0 days: ^∗^*p* < 0.05, ^∗∗^*p* < 0.01.

LPS was used to stimulate RAW 264.7 macrophages. The expression of ADAR1 was first increased and then decreased after day 2; this agrees with our previous observation that ADAR1 was increased within 24 h after LPS activation [10]. SOCS3 is a key molecule involved in the negative regulation of cytokines that signal through the JAK/STAT3 pathway. In the early stage, SOCS3 was activated in the macrophages, whereas at days 3 and 4, the expression of SOCS3 was decreased. The change in the protein level was consistent with the change of mRNA both in ADAR1 and SOCS3 (Figures 3(a) and 3(b)). Among all inflammatory factors, the levels of the proinflammatory factor IL-6 were increased most significantly over time (Figure 3(c)).

**Figure 4:**
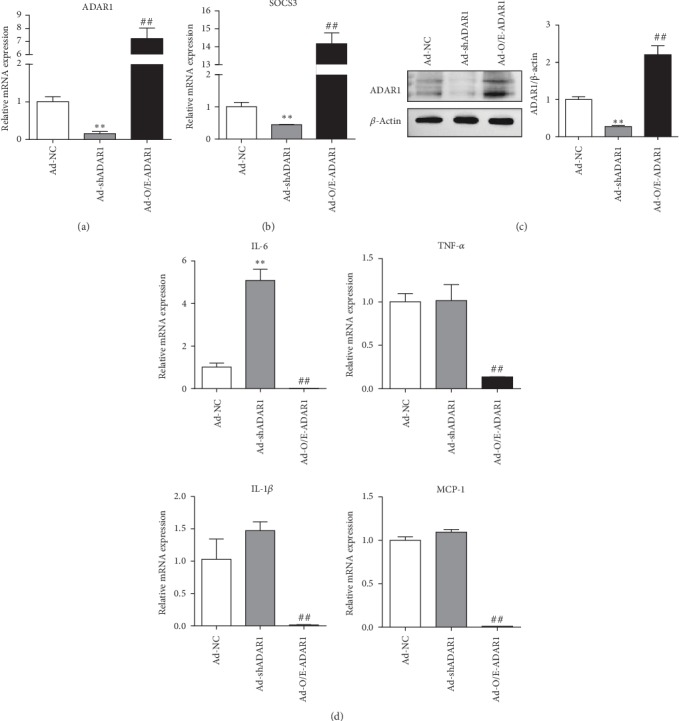
Effect of Ad-NC, Ad-shADAR1, and Ad-O/E-ADAR1 infections of RAW 264.7 macrophages on (a, c) mRNA and protein expression levels of ADAR1 and (b, d) mRNA expression levels of SOCS3, IL-6, TNF-*α*, IL-1*β*, and IL-10. Ad-shADAR1 vs. Ad-NC: ^∗^*p* < 0.05, ^∗∗^*p* < 0.01; Ad-O/E-ADAR1 vs. Ad-NC: ^#^*p* < 0.05, ^##^*p* < 0.01.

Given that ADAR1 can alleviate intestinal injury and inflammation in septic mice, we investigated whether ADAR1 can reduce inflammatory responses in macrophages. We transfected RAW 264.7 cells with Ad-NC, Ad-shADAR1, and Ad-O/E-ADAR1 and detected changes in the expression of inflammatory factors. After treatment with Ad-shADAR1, SOCS3 expression was decreased significantly (Figure 4(b), *p* = 0.002), whereas IL-6 expression was increased significantly (Figure 4(d), *p* < 0.001). After Ad-O/E-ADAR1 transfection, SOCS3 expression was increased significantly (Figure 4(b), *p* < 0.001), whereas the expression of IL-6 (*p* < 0.001), TNF-*α*, IL-1*β*, and MCP-1 was decreased (Figure 4(d)). SOCS3 expression was consistent with the changes in ADAR1 levels, whereas the opposite trend was observed for IL-6. As SOCS3 can inhibit the IL-6/STAT3 pathway, ADAR1 is expected to reduce IL-6 levels by regulating the expression of SOCS3, thereby inhibiting inflammation and alleviating sepsis.

### 3.4. ADAR1 Editing Enzyme Activity Regulated Biogenesis of miR-30a

**Figure 5:**
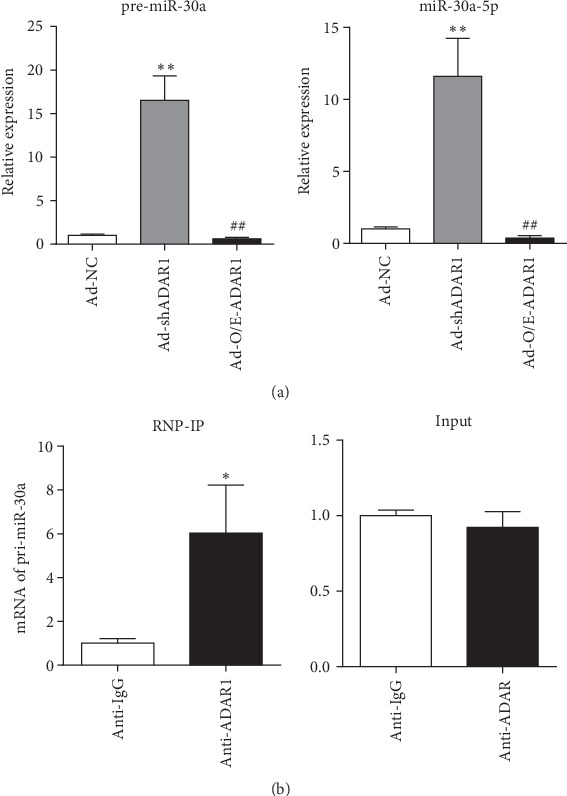
ADAR1 regulates miR-30a biogenesis. (a) Expression of pre-miR-30a and miR-30a-5p in RAW 264.7 cells infected with Ad-NC, Ad-shADAR1, and Ad-O/E-ADAR1 viruses. (b) Levels of pri-miR-30a RNA coimmunoprecipitated from RAW 264.7 mouse macrophage cell lysates with anti-GAPDH Ab (IgG control) and anti-ADAR1 Ab (left) and levels of total input pri-miR-30a (right). Ad-shADAR1 vs. Ad-NC: ^∗^*p* < 0.05, ^∗∗^*p* < 0.01; Ad-O/E-ADAR1 vs. Ad-NC: ^#^*p* < 0.05, ^##^*p* < 0.01.

We used Vienna-RNA Web to predict the potential interaction between ADAR1 and pri-miR-30a. Three adenoviruses (Ad-NC, Ad-shADAR1, and Ad-O/E-ADAR1) were used to transfect RAW 264.7 cells; changes in miR-30a expression in the transfectants were examined (Figure 5(a)). After transfection with Ad-shADAR1, the levels of pre-miR-30a (*p* < 0.001) and miR-30a-5p (*p* = 0.002) were increased significantly. After transfection with Ad-O/E-ADAR1, the levels of both pre-miR-30a (*p* < 0.001) and miR-30a-5p (*p* = 0.006) were decreased significantly. These results suggest that ADAR1 exerts a significant regulatory effect on miR-30a levels. RNP immunoprecipitation was performed to confirm whether ADAR1 performs A-to-I editing on pri-miR-30a (Figure 5(b)). The results of qRT-PCR confirmed the binding of ADAR1 to pri-miR-30a (*p* = 0.017), revealing that the regulatory action of ADAR1 was required for the biogenesis of pri-miR-30a.

### 3.5. miR-30a-5p Regulates SOCS3 Expression

**Figure 6:**
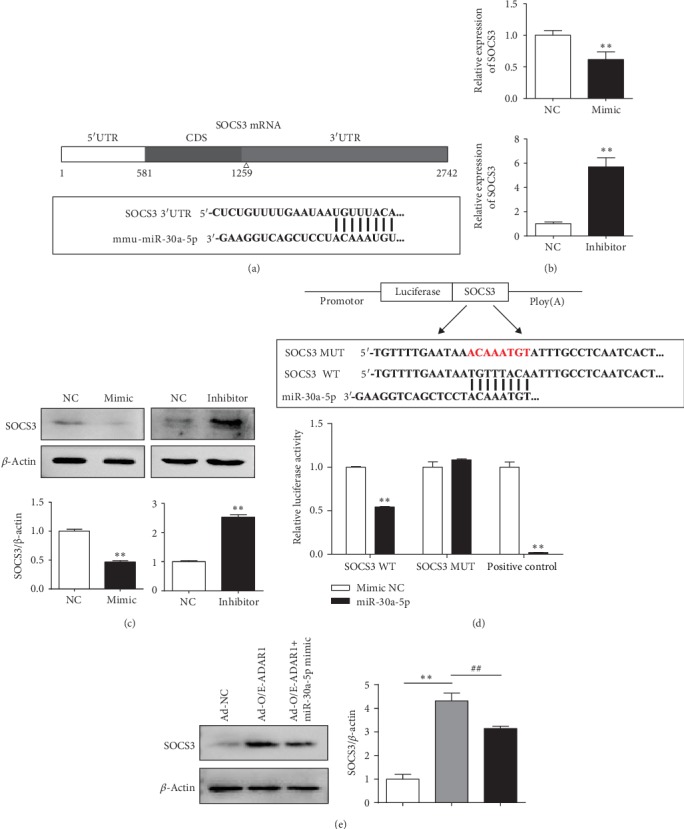
Regulation of SOCS3 expression by miR-30a-5p. (a) Predicted binding sites of miR-30a and SOCS3. (b, c) Expression of SOCS3 mRNA and protein after transfection of RAW 264.7 cells with miR-30a-5p mimic and inhibitor. (d) Luciferase activity of HEK 293T cotransfected with miR-30a-5p mimics and pmirGLO_PC, pmirGLO_SOCS3 MUT, or pmirGLO_SOCS3 WT. ^∗^*p* < 0.05, ^∗∗^*p* < 0.01. (e) Expression of SOCS3 mRNA and protein after transfection of RAW 264.7 cells with Ad-NC, Ad-O/E-ADAR1, and Ad-O/E-ADAR1+miR-30a-5p mimics. Ad-O/E-ADAR1 vs. Ad-NC: ^∗^*p* < 0.05, ^∗∗^*p* < 0.01; Ad-O/E-ADAR1+miR-30a-5p mimic vs. Ad-O/E-ADAR1: ^#^*p* < 0.05, ^##^*p* < 0.01.

**Figure 7:**
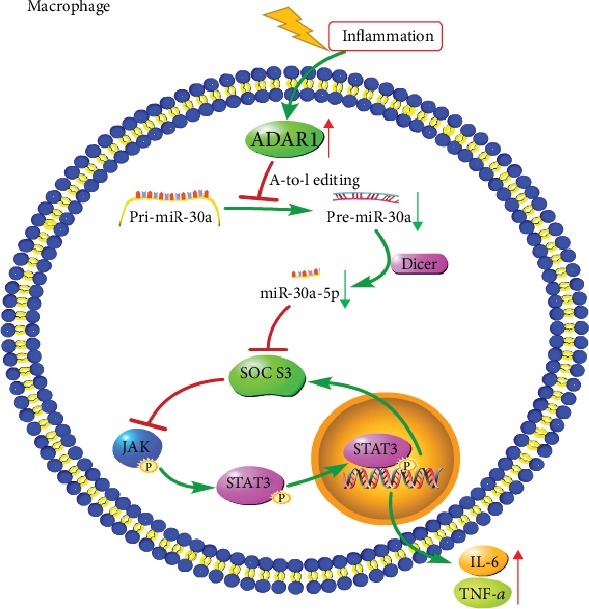
Using A-to-I editing, ADAR1 can reduce the maturation of miR-30a, upregulate SOCS3 expression, and then inhibit JAK/STAT3 signaling and reduce IL-6 and TNF-*α* expression.

We searched for miRNAs that bind to SOCS3 using the TargetScan database and related literature and identified miR-30a-5p as a highly expressed candidate that may regulate SOCS3 mRNA expression (Figure 6(a)). A mimic or inhibitor of miR-30a-5p was transfected into RAW 264.7 cells, after which the expression of SOCS3 mRNA and protein was measured by qRT-PCR and western blotting, respectively. SOCS3 expression was decreased (*p* < 0.01) after transfection with the mimic and increased (*p* < 0.001) after transfection with the inhibitor when compared with that in the NC group (Figures 6(b) and 6(c)); this result corroborates the finding that miR-30a-5p exerts a significant inhibitory effect on SOCS3 expression. We performed a luciferase assay to further confirm this direct interaction (Figure 6(d)). HEK 293T cells were cotransfected with miR-30a-5p mimics and pmirGLO_PC (positive control plasmid), pmirGLO_SOCS3 MUT, or pmirGLO_SOCS3 WT; luciferase activity in the transfectants was detected after 24 h of culture. The inhibition efficiency in the PC group (mmu-miR-30a-5p mimics+mirGLO_PC) was more than 90%. The negative control group (mmu-miR-30a-5p mimics+SOCS3 MUT) showed no significant inhibition of luciferase activity, whereas the experimental group (mmu-miR-30a-5p mimics+SOCS3 WT) showed an inhibition efficiency of approximately 50% (*p* < 0.001). This suggests that miR-30a-5p directly binds to SOCS3 (Figure 7).

To verify whether overexpression of ADAR1 affects SOCS3 via miR-30a, we transfected RAW 264.7 cells with Ad-NC, Ad-O/E-ADAR1, and Ad-O/E-ADAR1+miR-30a-5p mimics. SOCS3 protein (*p* < 0.001) expression was significantly increased after Ad-O/E-ADAR1 transfection but decreased (*p* = 0.004) after Ad-O/E-ADAR1 and miR-30a-5p mimic cotransfection (Figure 6(e)).

### 3.6. ADAR1-miR-30a-SOCS3 Axis-Mediated Regulation in the Murine Sepsis Model

**Figure 8:**
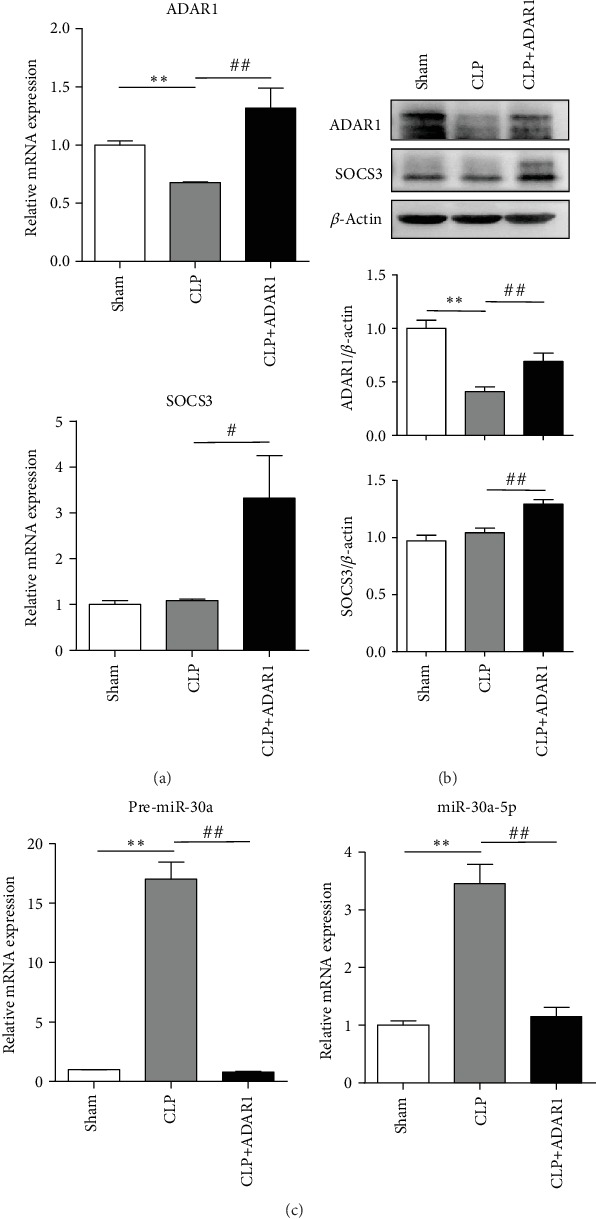
ADAR1 regulates miR-30a biogenesis and SOCS3 expression in mouse sepsis model. In a murine sepsis model, Ad-O/E-ADAR1 was injected 24 h after induction, the whole spleen was harvested and lysed, and mRNA and protein were extracted. (a) Expression of ADAR1 and SOCS3 was measured by qRT-PCR and western blotting, and (b) pre-miR-30a and miR-30a-5p were measured by qRT-PCR. CLP vs. sham: ^∗^*p* < 0.05, ^∗∗^*p* < 0.01; CLP+ADAR1 vs. CLP: ^#^*p* < 0.05, ^##^*p* < 0.01.

To verify the regulatory effect of ADAR1 on miR-30a maturation in the CLP model, the whole spleen was harvested and lysed in the CLP model, and mRNA and protein were extracted. In the CLP group, the expression of ADAR1 decreased significantly (Figures 8(a) and 8(b)); the pre-miR-30a (*p* < 0.001) and miR-30a-5p (*p* < 0.001) levels were significantly increased (Figure 8(c)) after Ad-O/E-ADAR1 administration. In contrast, in the CLP+ADAR1 group, the expression of ADAR1 and SOCS3 was increased significantly (Figures 8(a) and 8(b)), and pre-miR-30a (*p* < 0.001) and miR-30a-5p (*p* < 0.001) levels decreased significantly (Figure 8(c)) after Ad-O/E-ADAR1 administration. Figure 7 illustrates the hypothesis presented in this investigation.

## 4. Discussion

Our previous studies showed that overexpression of ADAR1 reduces the mortality of mice with infections [10]. We found that the levels of IL-6, TNF-*α*, IL-1*β*, and MCP-1 were significantly increased at 24 h after sepsis. In contrast, ADAR1 overexpression significantly reduced IL-6 levels in the spleen, consistent with IL-6 function as the main inflammatory factor, which attenuates the inflammatory response in sepsis that is inhibited by ADAR1. Previous studies have confirmed that SOCS3 is targeted by miR-30a [14, 15]. However, the specific mechanism by which ADAR1 reduces IL-6 expression and alleviates sepsis is not clear. We suspected that these effects are achieved by regulating the maturation of miRNA and expression of SOCS3.

This study demonstrated that ADAR1 ameliorates intestinal injury and inflammation via miR-30a-SOCS3-IL-6, suggesting that an ADAR1-miR-30a-SOCS3 axis downregulates the inflammatory response in septic mice.

In mammals, IL-6 is a pivotal modulator of the response to tissue damage caused by infection and disease [25], as it functions as both a proinflammatory and anti-inflammatory cytokine [26]. Because the IL-6 concentration is considered a biomarker for the diagnosis of sepsis, determination of accurate plasma IL-6 concentrations in the early stages of sepsis can be beneficial for providing adequate and timely therapeutic management of patients with severe symptoms, thereby reducing morbidity and mortality [27]. Studies have suggested that the measurement of IL-6 levels in the plasma from blood samples during the onset of sepsis can assist in the early identification of this condition and initiation of adequate treatment [28, 29]. The normal physiological range of IL-6 is 5–25 pg/mL, whereas in patients with sepsis, it may rapidly increase to 1,000 pg/mL. The serum concentration of IL-6 has been shown to be closely associated with the prognosis of sepsis; levels higher than 500 pg/mL were associated with 11% mortality, whereas levels > 7,500 pg/mL were associated with 37% mortality [29]. Therefore, treatment of sepsis by interfering with the involved signaling pathways is most effective in early stages [28].

During the inflammatory response, IL-6 induces the expression of acute phase protein and activates the JAK/STAT3 signaling pathway. STAT3 triggers multiple genes in response to cytokine (IL-6 family and IL-10) and growth factor stimulation, playing a critical role in anti-inflammatory/proinflammatory responses, cell growth, and cell death [12]. Upon activation, STAT3 translocates to the nucleus and induces the transcription of the corresponding *IL-6* response gene. Activated STAT3 also induces the expression of *SOCS3*. SOCS3 can specifically inhibit the IL-6 signal cascade and play an anti-inflammatory role in infectious diseases [22]. The key to this specificity is that SOCS3 directly interacts with gp130, the coreceptor of the IL-6 family of cytokines. This allows SOCS3 to specifically target the IL-6 signaling cascade [30, 31]. We found that SOCS3 protein expression was significantly increased after *ADAR1* overexpression. In RAW 264.7 cells after Ad-shADAR1 transfection, SOCS3 expression was decreased, and the IL-6 level was increased significantly. In contrast, after Ad-O/E-ADAR1 transfection, SOCS3 expression was increased significantly, and the expression of IL-6, TNF-*α*, IL-1*β*, and MCP-1 was decreased. These data suggest that ADAR1 reduces IL-6 levels and improves inflammation by regulating the expression of SOCS3.

miRNAs are 20–25-nucleotide endogenous noncoding RNAs with regulatory functions. They recognize target mRNAs via complementary base pairing, thereby degrading mRNA or inhibiting their translation. Many studies have demonstrated the important roles of miRNAs in the pathogenesis of sepsis [1]. They are first generated as primary miRNAs (pri-miRNAs) via RNA polymerase II-dependent transcription and then processed in the nucleus by Drosha-DGCR8 into pre-miRNA, with a ~70 nucleotide hairpin structure. These molecules are then exported to the cytoplasm via exportin-5, where they are further processed by Dicer to yield ~22-nucleotide miRNA duplexes. ADARs are thought to intervene at several steps during miRNA biogenesis [32]. ADAR1 can bind to Dicer, improve its cutting efficiency, and promote the production of mature miRNAs. ADAR1 can also perform A-to-I editing of pri-miRNAs to reduce the generation of corresponding mature miRNAs [5]. In viral myocarditis, ADAR1 p150 plays a key role in complexing with Dicer and promoting the expression of miRNA-222, the latter of which suppresses the expression of the target gene phosphatase-and-tensin (PTEN), which acts as a mediator of several cellular events including apoptosis, cell survival, proliferation, and migration [33]. In melanoma, loss of ADAR1 expression can lead to abnormal miRNA processing or functioning and changes in the total mature levels of miR-455-5p, contributing to melanoma growth and metastasis via downregulation of the tumor suppressor gene CPEB1 [34].

In the context of the inflammation regulation, miR-146a, miR-146b, and miR-155 exert anti-inflammatory properties by downregulating IL-6 and IL-8, as well as by affecting the expression of HSP10 in activated endothelium [35]. Among these functions, induction of miR-146a expression in splenic macrophages prevents excessive inflammation and sepsis-induced multiple organ injury [36]; miR-151-3p inhibits LPS-induced IL-6 production by targeting STAT3 [37], miR-142-3p, and miR-let-7g increase IL-6 production in neonatal polymorphonuclear leukocytes induced via posttranscriptional inhibition of LPS [38], and miR-19a enhances JAK-STAT signaling by regulating SOCS3 expression [39]. Moreover, miRNAs contribute to oncogenesis as both tumor suppressors and oncogenes and have therefore been widely studied as potential biomarkers for diagnosis, subtype classification, prognosis, and therapy in multiple cancers [40]. Downregulation of *miR-340* inhibited gastric cancer cell proliferation, arrested the cell cycle, and facilitated apoptosis by upregulating *SOCS3* to suppress the JAK-STAT3 signaling pathway [41]. Downregulation of *miR-221* may promote the expression of its target gene *SOCS3*, which inhibits the proliferation, invasion, and migration of hepatocellular carcinoma cells by repressing the JAK-STAT3 signaling pathway [42].

*miR-30a* is located on chromosome 6q13 and plays vital roles in many biological processes involved in cancer and inflammation regulation. It is downregulated in breast cancer, liver cancer, and oral cancer and has been suggested to function as a tumor suppressor [43]. Studies have shown that miR-30a-5p has an inhibitory effect on the occurrence and metastasis of non-small-cell lung cancer [43]. Experiments have shown that miR-30a-5p can regulate the occurrence and development of allergic disease by targeting the inhibition of *SOCS3* expression [13]. Overexpression of miR-30a can specifically inhibit *SOCS3* expression and enhance myeloid-derived suppressor cell differentiation and immunosuppression in mice with B-cell lymphoma [14]. However, downregulation of *miR-30a* can inhibit the JAK/STAT3 pathway by targeting *SOCS3* and reducing the tumor-forming ability of glioma stem cells [15]. In our study, the RNP-immunoprecipitation assay revealed that ADAR1 editing enzyme activity regulates the biogenesis of miR-30a, and the dual-luciferase reporter assay showed that miR-30a-5p, the mature form of miR-30a, targets SOCS3. While SOCS3 can inhibit the IL-6/STAT3 pathway, regulating the maturation of miR-30a can indirectly regulate the expression of IL-6.

## 5. Conclusions

We examined the therapeutic effect of ADAR1 on sepsis-related intestine injury. Administration of ADAR1-overexpressing adenovirus in septic mice was found to notably improve intestinal permeability. The mechanism of protection, at least in part, was due to a decrease in systematic inflammation mediated by macrophages. At present, it is difficult to inhibit the cascade of inflammatory factors involved in the acute stage of sepsis. This study revealed a potential protective effect of ADAR1 against inflammation in the acute stage of sepsis. However, further experimental studies are needed to explore the mechanisms and clinical applications of ADAR1 treatment.

## Figures and Tables

**Table 1:** Quantitative RT-PCR primers used in this research.

Gene	Forward (5′-3′)	Reverse (5′-3′)
ADAR1	CCGTACCATGTCCTGTAGTGACA	GCCCTTGGCTGAAAAGGTAAC
SOCS3	TCGCCACCTACTGAACCCTC	TGGTCCAGGAACTCCCGAAT
IL-1*β*	CAGGATGAGGACATGAGCACC	CTCTGCAGACTCAAACTCCAC
IL-4	ACCAGGAGCCATATCCAC	TTGGAAGCCCTACAGACG
IL-6	TGCTGGTGACAACCACGGC	GTACTCCAGAAGACCAGAGG
IL-10	GCCAGAGCCACATGCTCCTA	GATAAGGCTTGGCAACCCAAGTAA
TNF-*α*	CGTCAGCCGATTTGCTATCT	CGGACTCCGCAAAGTCTAAG
pri-miR-30a	GCGTGTAAACATCCTCGAC	GTGCAGGGTCCGAGGT
GAPDH	TGTGTCCGTCGTGGATCTGA	TTGCTGTTGAAGTCGCAGGAG

## Data Availability

The data used to support the findings of this study are available from the corresponding authors upon request.
